# Lung damage created by high tidal volume ventilation in rats with monocrotaline-induced pulmonary hypertension

**DOI:** 10.1186/s12890-022-01867-6

**Published:** 2022-03-05

**Authors:** Masako Kawai, Erquan Zhang, Jane Chanda Kabwe, Amphone Okada, Junko Maruyama, Hirofumi Sawada, Kazuo Maruyama

**Affiliations:** 1 Department of Anesthesiology and Critical Care Medicine, Mie University School of Medicine, 2-174 Edobashi, Tsu, Mie 514-8507 Japan; 2 Faculty of Medical Engineering, Suzuka University of Medical Science, 1001-1 Kishioka, Suzuka, Mie 510-0293 Japan; 3 Neonatology, Fuzhou Children’s Hospital of Fujian Medical University, 145-817 Middle Road, Gulou, Fuzhou, 350005 Fujian China; 4 Department of Pediatrics, Mie University School of Medicine, 2-174 Edobashi, Tsu, Mie 514-8507 Japan

**Keywords:** Ventilator-induced lung injury, Monocrotaline, Mechanical ventilation, Pulmonary hypertension

## Abstract

**Background:**

Rats with chronic hypoxia-induced non-inflammatory pulmonary hypertension (PH) are resistant to ventilator-induced lung injury. We investigated the effect of high tidal volume ventilation in another model of PH, monocrotaline (MCT)-induced PH, which is a type of inflammatory PH.

**Methods:**

PH was induced in rats by subcutaneous injection with 60 mg/kg MCT. Normal control rats, rats at 2 weeks after MCT injection (MCT2), and rats at 3 weeks after MCT injection (MCT3) were ventilated with low tidal volume (LV, 6 mL/kg) or high tidal volume (HV, 35 mL/kg) for 2 h with room air without positive end-expiratory pressure. Arterial oxygen pressure (PaO_2_) and Evans blue dye (EBD) extravasation were measured. Hypertensive pulmonary vascular remodeling was assessed morphometrically by the percentage of muscularized peripheral pulmonary arteries (%Muscularization) and the media wall thickness to external diameter ratio, namely percentage medial wall thickness (%MWT). To assess inflammation, lung IκB protein and cytokine mRNA expression levels were assessed.

**Results:**

Baseline mean pulmonary arterial pressure was significantly higher in MCT rats (normal, 15.4 ± 0.5 mmHg; MCT2, 23.7 ± 0.9; and MCT3, 34.5 ± 1.5). After 2-h ventilation, PaO_2_ was significantly lower in the HV groups compared with the LV groups in normal and MCT2 rats, but not in MCT3 rats. Impairment of oxygenation with HV was less in MCT3 rats compared with normal and MCT2 rats. Among the HV groups, MCT3 rats showed significantly lower levels of EBD extravasation than normal and MCT2 rats. HV significantly downregulated IκB protein expression in normal and MCT3 rats and increased IL-6, MCP-1, CXCL-1 (MIP-1), and IL-10 mRNA levels in MCT3 rats. %Muscularization, %MWT, and the expression of lung elastin were significantly higher in MCT3 rats than in normal and MCT2 rats.

**Conclusion:**

We found that HV-associated damage might be reduced in MCT-induced PH rats compared with normal rats. The results of this and earlier studies suggest that hypertensive pulmonary vascular structural changes might be protective against the occurrence of ventilator-induced lung injury, irrespective of the etiology of PH.

**Supplementary Information:**

The online version contains supplementary material available at 10.1186/s12890-022-01867-6.

## Background

Mechanical ventilation is used under general anesthesia in patients with and without lung disease and patients with respiratory failure. Injurious ventilation, such as high tidal volume ventilation, might worsen preexisting lung damage or initiate new lung injury in healthy lungs, termed ventilator-induced lung injury (VILI) [1–6], which is associated with the release of inflammatory mediators. The induction of an inflammatory response with VILI is evidenced by neutrophil and macrophage infiltration of the lung [7, 8] and increases in tumor necrosis factor (TNF) α, interleukin (IL)-1α, IL-1β, IL-6, and MIP-2 levels in bronchoalveolar lavage fluid [9–11] and plasma [9, 10] and lung mRNA expression [11].

Pulmonary hypertension (PH) occurs in patients with congenital heart disease with a left-to-right shunt [12–15], chronic obstructive lung disease [16], pulmonary fibrosis [17], and connective tissue disease [18, 19]. Some patients with these conditions may undergo surgery under general anesthesia with mechanical ventilation, and postoperative complications can lead to prolonged mechanical ventilation. In all conditions causing PH in humans and experimental models of PH vascular remodeling, there is new muscularization of normally non-muscular peripheral pulmonary arteries, medial wall hypertrophy of muscular arteries, and increased protein expression in vascular connective tissue [12, 15, 20, 21]. Chronic hypoxia-induced PH [22–27] and monocrotaline (MCT)-induced PH [28–34] in rats are the most commonly used animal models of PH [18, 19, 21, 35], and both models undergo vascular remodeling [15, 20, 21]. In MCT-induced PH, endothelial changes precede the rise of pulmonary artery pressure, which is associated with inflammatory changes, as evidenced by neutrophil and monocyte infiltration and increased inflammatory cytokine expression in the lung, such as IL-1, TNFα, and monocyte chemotactic protein (MCP)-1 [18, 28–30]. Kornecki et al. showed that vascular remodeling in chronic hypoxia-induced PH rats protects against the effects of injurious mechanical ventilation in vivo [27]. Thus, this vascular remodeling might have an effect on the development of VILI. Although vascular remodeling is associated with MCT-induced PH, high tidal volume ventilation might exacerbate lung injury given that MCT-induced PH has an inflammatory component in its etiology [18, 19, 28–30]. Therefore, in the present study, we compared the effects of high tidal volume ventilation in normal rats and MCT-induced PH rats.

## Methods

### Animals

The Animal Experiments Committee of the Mie University School of Medicine, Mie, Japan approved the study protocol (approval no. 30-13). Seven-week-old male Sprague–Dawley rats (Japan SLC, Inc.) were subcutaneously injected with 60 mg/kg MCT to induce PH [30, 31, 33], and kept for 2 or 3 weeks. Rats at 2 weeks after MCT injection (MCT2) were defined as a mild PH model, whereas those at 3 weeks after MCT injection (MCT3) were defined as a severe PH model. Age-matched untreated normal rats were used as a normal group. The total number of rats used in this study was 103. For euthanasia rats were administered 50 mg/kg pentobarbital sodium (Somuno Pentil injection^®^, Kyoritsu Seiyaku Corporation, Tokyo, Japan) via intraperitoneal injection. After obtaining no consciousness with respiratory depression and no response to the stimulation, which took about 5–10 min and showed deep anesthesia, rats were put under mechanical ventilation through tracheostomy. Then the abdomen was incised and the rats were exsanguinated by aortic incision and heart and lung samples were removed.

### Experimental groups

Eighty-five rats were randomly assigned to one of six groups: (1) rats ventilated with low tidal volume of 6 mL/kg (normal/LV) (n = 14); (2) rats ventilated with high tidal volume of 35 mL/kg (normal/HV) (n = 22); (3) rats ventilated with low tidal volume of 6 mL/kg at 2 weeks after a single subcutaneous injection of 60 mg/kg MCT (MCT2/LV) (n = 11); (4) rats ventilated with high tidal volume of 35 mL/kg at 2 weeks after a single subcutaneous injection of 60 mg/kg MCT (MCT2/HV) (n = 15); (5) rats ventilated with low tidal volume of 6 mL/kg at 3 weeks after a single subcutaneous injection of 60 mg/kg MCT (MCT3/LV) (n = 11); and (6) rats ventilated with high tidal volume of 35 mL/kg at 3 weeks after a single subcutaneous injection of 60 mg/kg MCT (MCT3/HV) (n = 12). The number of rats used in each experiment are presented in each figure. Rats with mean pulmonary arterial pressure less than 25 mmHg in the MCT3/LV and MCT3/HV group were excluded. Since pulmonary hypertension is a condition defined on condition that the mean pulmonary arterial pressure at rest is 25 mmHg or higher, rats with a mean pulmonary arterial pressure of less than 25 mmHg were excluded in order to examine respiratory management for MCT3 PH rats, ie, severe PH in the present study.

### Catheterization

Under intraperitoneal pentobarbital sodium (45 mg/kg) anesthesia, a right internal carotid and a pulmonary artery catheter (Silastic tubing, 0.31 mm inner diameter and 0.64 mm outer diameter) were inserted by using the closed-chest technique, as described previously [11, 23, 30, 33]. Mean pulmonary arterial pressure (mPAP), and peak inspiratory airway pressure (PIP) at the tracheostomy cannula (SP-110; Natsume, Tokyo, Japan) were recorded with a physiological transducer and amplifier system (AP 620; Nihon Kohden, Tokyo, Japan) [11, 13, 24, 25, 30, 33].

#### Mechanical ventilation

The rats were ventilated without positive end-expiratory pressure using an SN-480-7 volume cycle ventilator (Shinano Co., Tokyo, Japan) [11]. All groups of rats were ventilated for 2 h with room air. The respiratory rate and dead space were adjusted by inserting a tube between the Y-piece of the ventilator circuit and tracheostomy cannula in the high tidal volume groups [11], so that arterial CO_2_ tension was kept between 40–50 mmHg.

### Experimental protocol

#### Arterial oxygen pressure

After 15 min stabilization of ventilation with a tidal volume of 6 mL/kg, baseline arterial blood gas analysis was performed by a portable blood gas analyzer (iSTAT Analyzer 200; Abbott Point of Care, Inc., Princeton, NJ), which was followed by experimental tidal volume (6 or 35 mL/kg) assignment [11] with continuation of mechanical ventilation for another 2 h. Further arterial blood samples were obtained at 0.5, 1.0, 1.5, and 2.0 h after tidal volume assignment. For the measurement of Evans blue dye (EBD) extravasation, 30 mg/kg EBD was given intravenously after blood sampling at 0.5 h after tidal volume assignment. After 2-h ventilation, tidal volume was returned to 6 mL/kg and PIP was measured.

### Preparation of lung tissue for determining the percentage of lung water content and EBD extravasation

At the end of the protocol, lung tissue was obtained to measure lung water content and pulmonary microvascular permeability [11, 33]. The upper lobe of the right lung was used for the measurement of the percentage of lung water content, (wet lung weight − dried lung weight)/(wet lung weight) × 100. The rest of the lung was used to determine EBD extravasation into the lung, as an estimate of protein permeability, which was quantitated as described previously [11, 35–37].

### Right ventricular hypertrophy

To estimate right ventricular hypertrophy (RVH), the heart was removed and fixed in buffered neutral formaldehyde solution [23, 33]. The right ventricle (RV) of the heart was dissected from the left ventricle plus septum (LV + S) and weighed separately. The heart weight ratio (RV/[LV + S]) was calculated to assess RVH [23, 30, 33].

### Lung samples for western blotting, real-time PCR, and vascular structural assessment

For western blotting and real-time PCR analyses of the lung, the hilum of the right lung was ligated and the right lung was excised and put into liquid nitrogen. To assess hypertensive pulmonary vascular structural changes, the left lung was injected with a hot barium-gelatin mixture at a pressure of 36 cmH_2_O and fixed in 10% formalin for 72 h, as described previously [23–25, 30, 33]. Lung sections were stained for elastin by the Van Gieson method [23–25, 30, 33].

### Morphometry of the pulmonary vasculature

To assess the magnitude of hypertensive pulmonary vascular remodeling, light microscope (Olympus CX33, Tokyo, Japan) slides were analyzed at ×400, without previous knowledge of the treatment group, which was described previously [23–26, 30, 33]. The acquisition resolution of the microscope image was 1920 × 1440 pixels. The percentage of muscularized arteries (%Muscularization) in peripheral pulmonary arteries with an external diameter of 15–50 μm and 51–100 μm and the ratio of media wall thickness (distance between the external and internal elastic laminae) to the external diameter in small muscular artery, namely percentage medial wall thickness (%MWT), were calculated [24, 25, 30]. The number of arteries were 445 arteries per section (166–634 arteries per rat). An average 33 (0–75 per section) small muscular arteries with a diameter of 51–100 μm and an average 16 (2–30 arteries per section) small muscular arteries with a diameter of 101–200 μm were analysed, respectively.

### Western blotting for IκB, high-mobility group box 1, and elastin

Western blotting for IκB, high-mobility group box 1 (HMGB-1), and elastin in whole lung tissue was performed as described previously [11, 24, 25]. The following primary antibodies were used: anti-HMGB-1 (1:5,000; #3935S; Cell Signaling Technology, Danvers, MA); anti-IκBα (1:5,000 dilution; #4814S; Cell Signaling Technology); anti-elastin (1:5,000 dilution; 15257-1-AP; Proteintech, Rosemont, IL); and anti-β-actin (1:200,000 dilution; A5441; Sigma-Aldrich, St. Louis, MO). The following secondary antibodies were used: anti-mouse IgG-HRP (1:20,000 dilution; NA 931; Amersham, Piscataway, NJ) and anti-rabbit IgG-HRP (1:20,000 dilution; D2313; Santa Cruz Biotechnology, Dallas, TX). The immunoreactive bands were visualized with ECL reagents in AlphaView software 3.3.0 (ProteinSimple, San Jose, CA).

### TaqMan real-time PCR

The TaqMan Gene Expression Assay was used for analysis of IL-6 (Rn01410330), IL-10 (Rn01644839), C-X-C motif chemokine ligand 1 (CXCL-1/KC/MIP-1) (Rn00578225), MCP-1 (Rn00580555), HMGB-1 (Rn02377062), Elastin (Rm01499782) and β-actin (Rn00667869), as described previously [24].

### BALF sampling

In the other sets of normal, MCT2 and MCT3 rats (n = 5, respectively), bronchoalveolar lavage (BAL) was performed to show the magnitude of lung inflammation without mechanical ventilation. The rats were anesthetized by the intraperitoneal administration of 50 mg/kg pentobarbital sodium. The BAL fluid (BALF) was obtained by cannulating the trachea with plastic tube and by infusing the lungs with 7-ml of PBS. The recovered fluid (BALF) was centrifuged (2200 rpm, 10 min) using a cytofuge (Medite, Germany), and the cells were stained with May Grunwald Giemsa (Merck, Darmstadt, Germany).

### Data analysis

All values are expressed as the mean ± standard error. Analysis of variance with repeated measures was used for arterial blood gas, hemodynamic and PIP analysis. EBD extravasation, the percentage of lung water content, right ventricular hypertrophy, cytokine and chemokine mRNA levels, IκB and HMGB-1 protein expressions were performed by two-way analysis of variance. Morphologic parameters and elastin protein expression levels were  performed by one-way analysis of variance. When significant variance was found, Fisher’s predicted least significant difference test was used to establish which groups were different. *p* < 0.05 was considered to be significant.

## Results

### Arterial oxygen pressure

#### Baseline

**Fig. 1:**
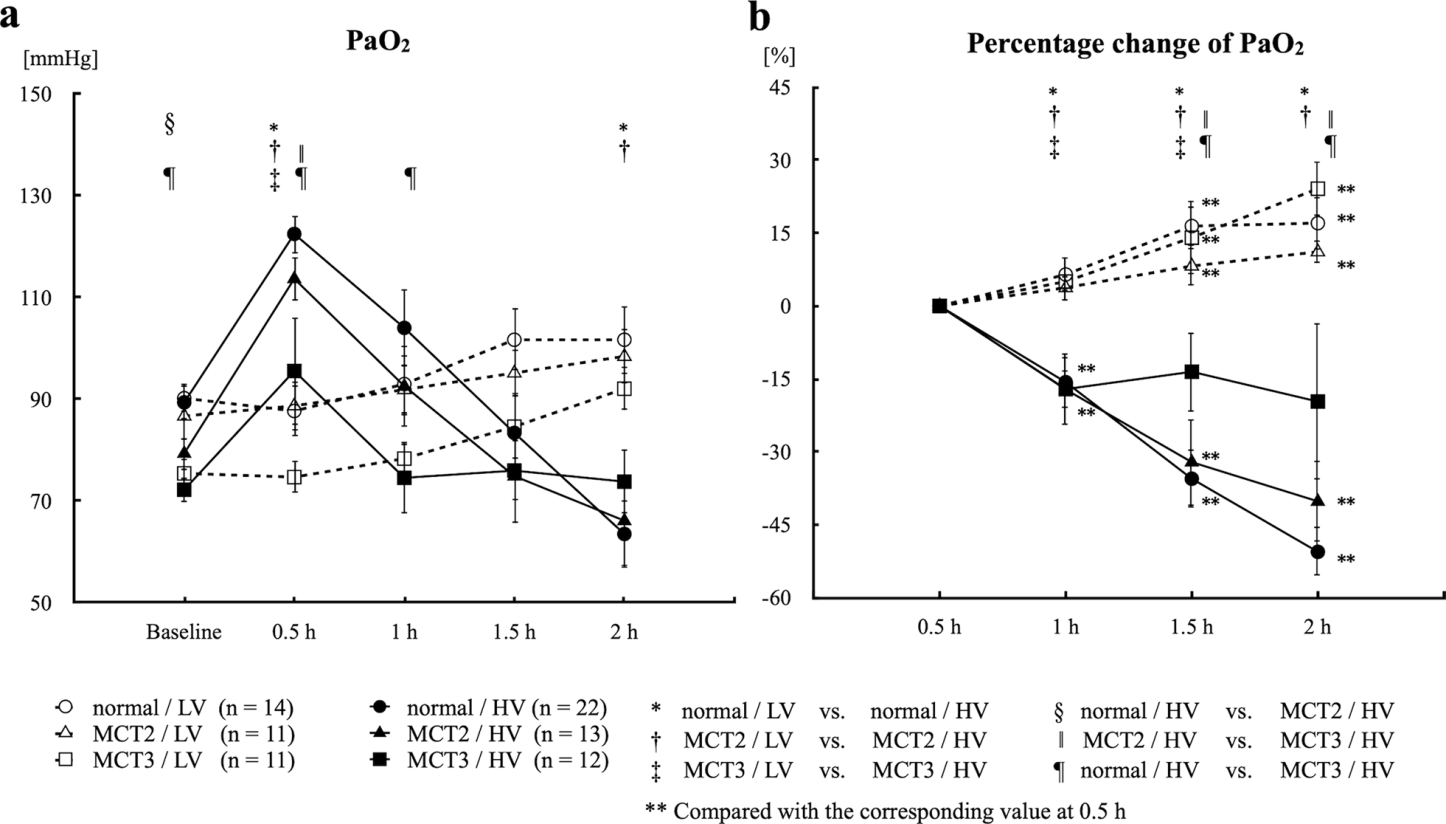
Arterial oxygen pressure (PaO_2_) and percentage change of PaO_2_. **a** PaO_2_. **b** Percentage change of PaO_2_ at 1, 1.5, and 2 h, taking the value at 0.5 h as a reference. MCT2: rats at 2 weeks after monocrotaline injection; MCT3: rats at 3 weeks after monocrotaline injection; LV: low tidal volume (6 mL/kg); HV: high tidal volume (35 mL/kg); Baseline: immediately after the assignment of experimental tidal volume; 0.5, 1, 1.5, and 2 h: time after the assignment of experimental tidal volume (6 or 35 mL/kg). Bars indicate mean ± standard error. n = number of rats

There were no significant differences in baseline arterial oxygen (PaO_2_) between the LV and HV groups in normal, MCT2, and MCT3 rats before the administration of high tidal volume ventilation (Fig. 1).

#### At 2 h after the start of high tidal volume ventilation

After 2-h ventilation, PaO_2_ was significantly lower in the HV groups compared with the LV groups in normal and MCT2 rats, but not in MCT3 rats (Fig. 1a). Given that PaO_2_ increased and peaked at 0.5 h after the start of high tidal volume ventilation and then declined in the HV groups, we calculated the percentage decrease of PaO_2_ at 1, 1.5, and 2 h, taking the value at 0.5 h as a reference. The percentage decrease of PaO_2_ in the HV groups at 2 h was significantly less in MCT3 rats compared with normal and MCT2 rats (Fig. 1b). These results suggested that the impairment of oxygenation with high tidal volume ventilation was less in MCT3 rats compared with normal and MCT2 rats.

### EBD extravasation and the percentage of lung water content

**Fig. 2:**
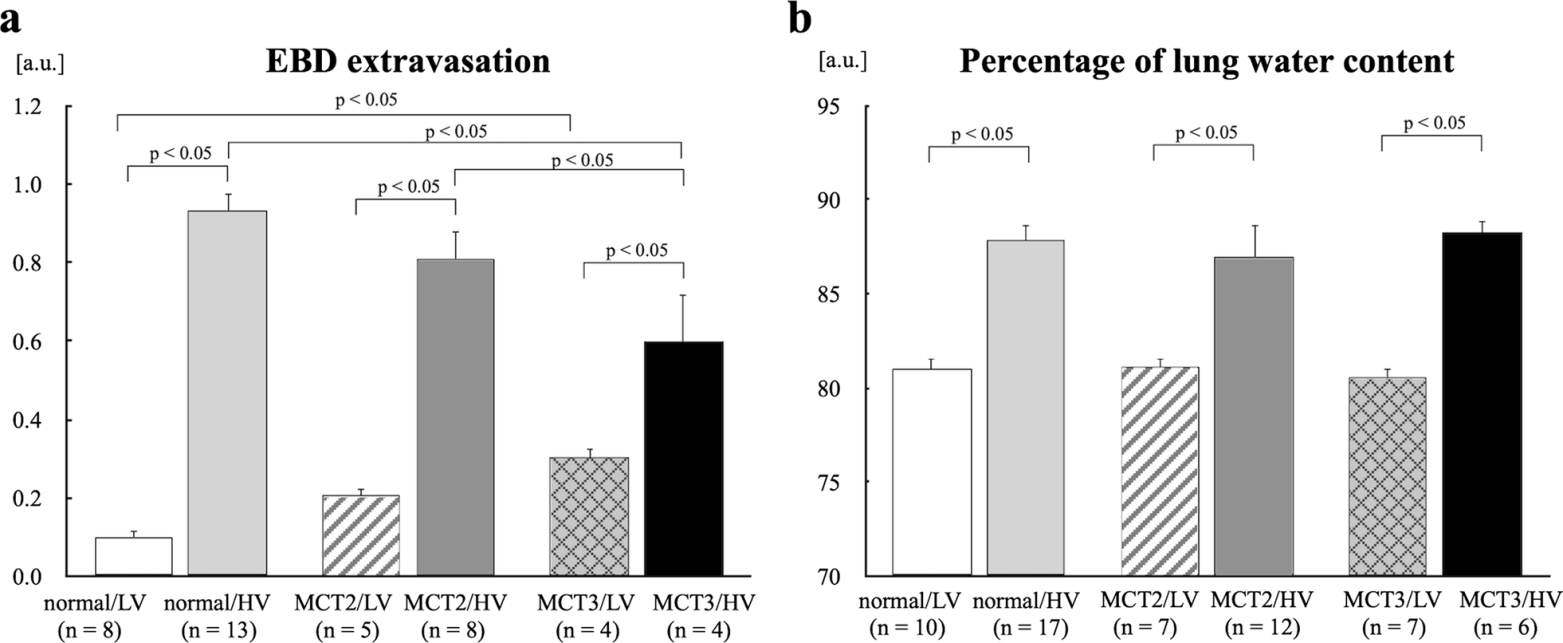
Evans blue dye (EBD) extravasation and the percentage of lung water content. **a** EBD extravasation, an estimate of protein permeability. **b** The lung dry-to-wet weight ratio, an estimate of lung water content. MCT2: rats at 2 weeks after monocrotaline injection; MCT3: rats at 3 weeks after monocrotaline injection; LV: low tidal volume (6 mL/kg); HV: high tidal volume (35 mL/kg); a.u.: arbitrary units. Bars indicate mean ± standard error. n = number of rats

EBD extravasation, a marker of protein permeability, was significantly increased in all HV groups compared with the corresponding LV groups (Fig. 2a). Among the HV groups, MCT3 rats showed significantly lower EBD extravasation than normal and MCT2 rats. In contrast, among the LV groups, MCT3 rats showed significantly higher EBD extravasation than normal rats (Fig. 2a). The percentage of lung water content was increased in all HV groups compared with the corresponding LV groups in normal, MCT2, and MCT3 rats (Fig. 2b). There were no significant differences in the percentage of lung water content among the HV groups (Fig. 2b).

### Mean pulmonary arterial pressure

#### Baseline

**Fig. 3:**
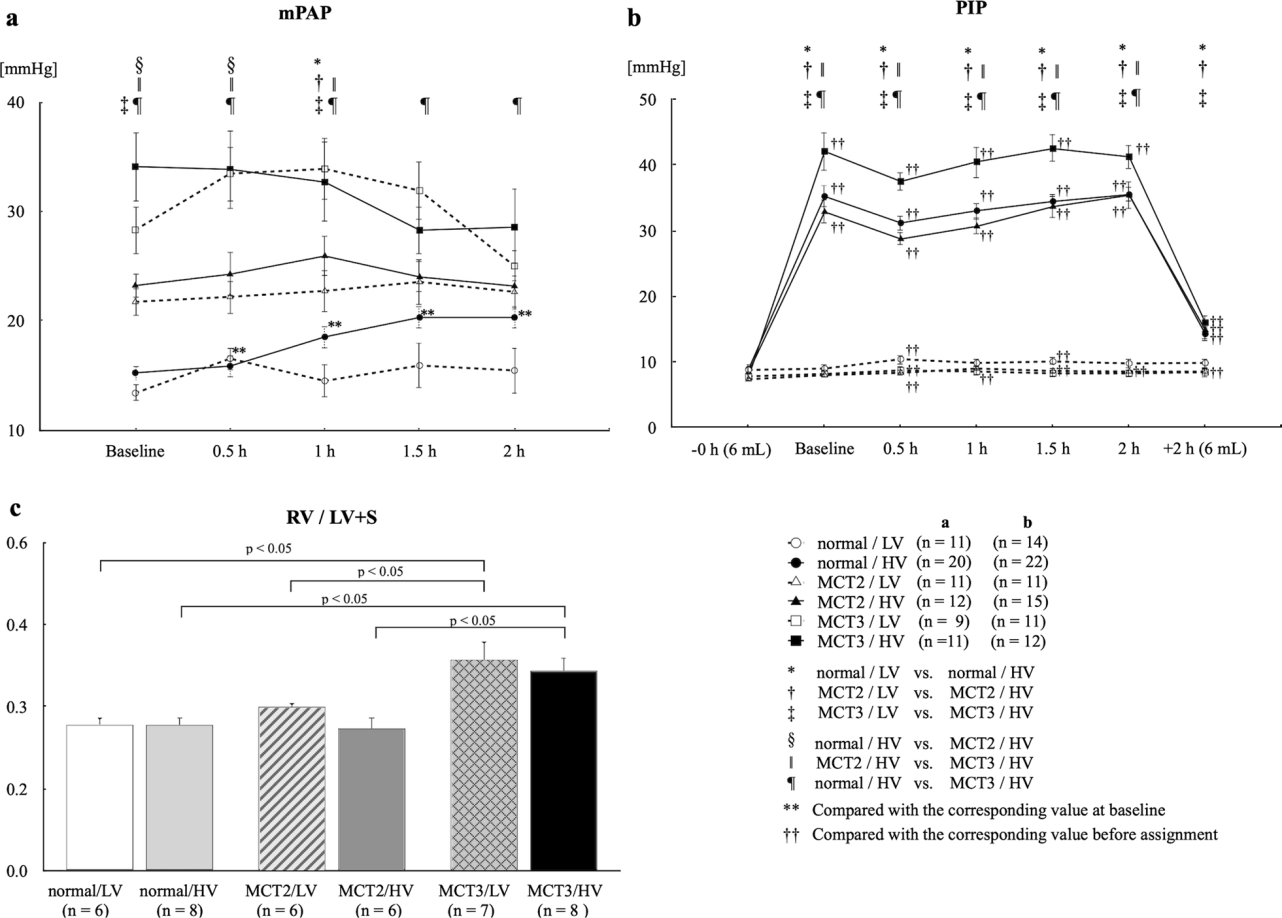
Mean pulmonary arterial pressure (mPAP), peak inspiratory airway pressure (PIP), and RV/ (LV + S) ratio. **a** mPAP. **b** PIP. **c** right ventricular weight (RV)/left ventricular plus septal weight (LV + S) ratio (RV/[LV + S] ratio), an estimate of right ventricular hypertrophy. MCT2: rats at 2 weeks after monocrotaline injection; MCT3: rats at 3 weeks after monocrotaline injection; LV: low tidal volume (6 mL/kg); HV: high tidal volume (35 mL/kg); Baseline: immediately after the assignment of experimental tidal volume; 0.5, 1, 1.5, and 2 h: time after assignment of experimental tidal volume (6 or 35 mL/kg); −0 h (6 mL) (**b**): PIP before the assignment of experimental tidal volume while all rats were ventilated with a tidal volume of 6 mL/kg; + 2 h (6 mL) (**b**): PIP when returning to 6 mL/kg from 35 mL/kg after the end of 2-h ventilation. Bars indicate mean ± standard error. n = number of rats

Baseline mPAP was significantly higher in MCT2 and MCT3 rats than in normal rats (mPAP: normal, 15.4 ± 0.5 mmHg; MCT2, 23.7 ± 0.9 mmHg; and MCT3, 34.5 ± 1.5 mmHg) (Fig. 3a). This increase in PAP was associated with a significant increase in RVH in MCT3 rats (Fig. 3c), suggesting the development of PH.

#### Changes of mPAP during mechanical ventilation

Changes of mPAP were analyzed taking the value at baseline as a reference to detect the effect of HV ventilation, because this time point was the first time point after the assignment of high tidal volume ventilation. There were no time-dependent changes in mPAP in any of the LV groups. In normal rats, HV ventilation significantly increased mPAP at 0.5, 1, 1.5, and 2 h after the start of mechanical ventilation compared with baseline (Fig. 3a), whereas there were no changes in MCT2 and MCT3 rats in the HV groups.

### Peak inspiratory airway pressure

PIP was not changed in any of the LV groups throughout the course of the experiment. In contrast, PIP was increased in all HV groups after the assignment of HV until the end of HV mechanical ventilation. MCT3 rats had significantly higher PIP compared with MCT2 rats (Fig. 3b), suggesting a decrease in lung compliance in MCT3 rats. When the tidal volume in the HV groups was returned to the low volume of 6 mL/kg after the end of 2-h ventilation, PIP was reduced, but it was significantly higher than the values prior to 0 h in all normal, MCT2, and MCT3 rats, suggesting decreased lung compliance after 2 h in the HV groups.

### Vascular structural remodeling

**Fig. 4:**
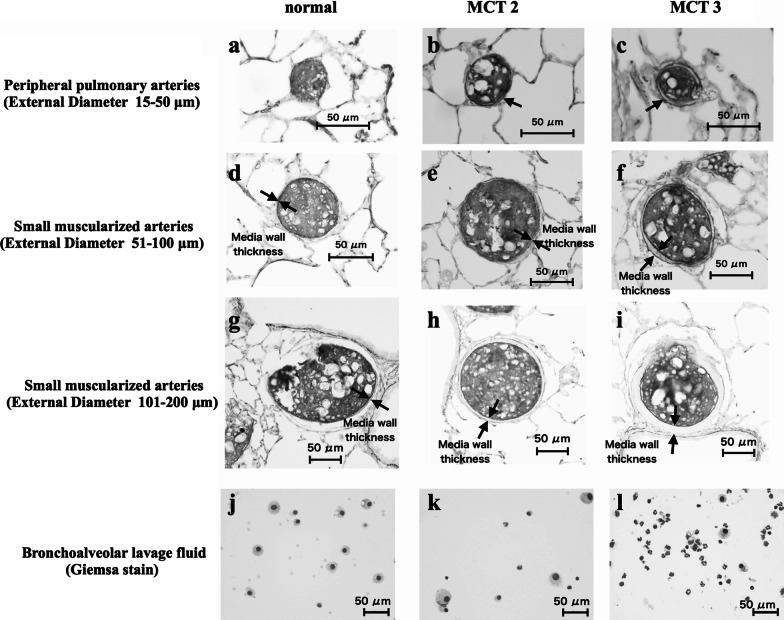
Light micrographs of peripheral pulmonary arteries and muscularized arteries. **a**, **b**, **c** Peripheral pulmonary arteries. There was no apparent muscle layer in the peripheral pulmonary arteries of normal rats (**a**). Monocrotaline (MCT)-induced new muscularization of normally non-muscularized arteries (**b**, **c**). Arrows **b**, **c** denote new muscularization in MCT rats. **d**, **e**, **f** Muscularized arteries with an external diameter of 51–100 μm. **g**, **h**, **i** Muscularized arteries with an external diameter of 101–200 μm. **e**, **f**, **h**, **i** MCT-induced medial hypertrophy of small muscularized arteries. Medial wall thickness was high in small muscularized arteries with an external diameter of 101–200 μm **i** in MCT3 rats. Arrows denote the muscle layer. **j**, **k**, **l** The numbers of macrophages and neutrophils in MCT-injected rats were increased in MCT2 (**k**) and MCT3 (**l**) rats as compared with the normal group (**j**). MCT2: rats at 2 weeks after MCT injection; MCT3: rats at 3 weeks after MCT injection

Representative arteries are shown in Fig. 4, demonstrating new muscularization of peripheral pulmonary arteries (Fig. 4b, c) and hypertrophy of the media in small muscular arteries (Fig. 4e, f, h, i). To evaluate hypertensive pulmonary vascular remodeling in the MCT2 and MCT3 groups, %Muscularization and %MWT were assessed. The data from the low and high tidal volume groups were combined. To show the inflammatory responses in the lung representative slides of BALF sample are also shown (Fig. 4j, k, l).

#### Percentage of muscularized peripheral arteries

**Fig. 5:**
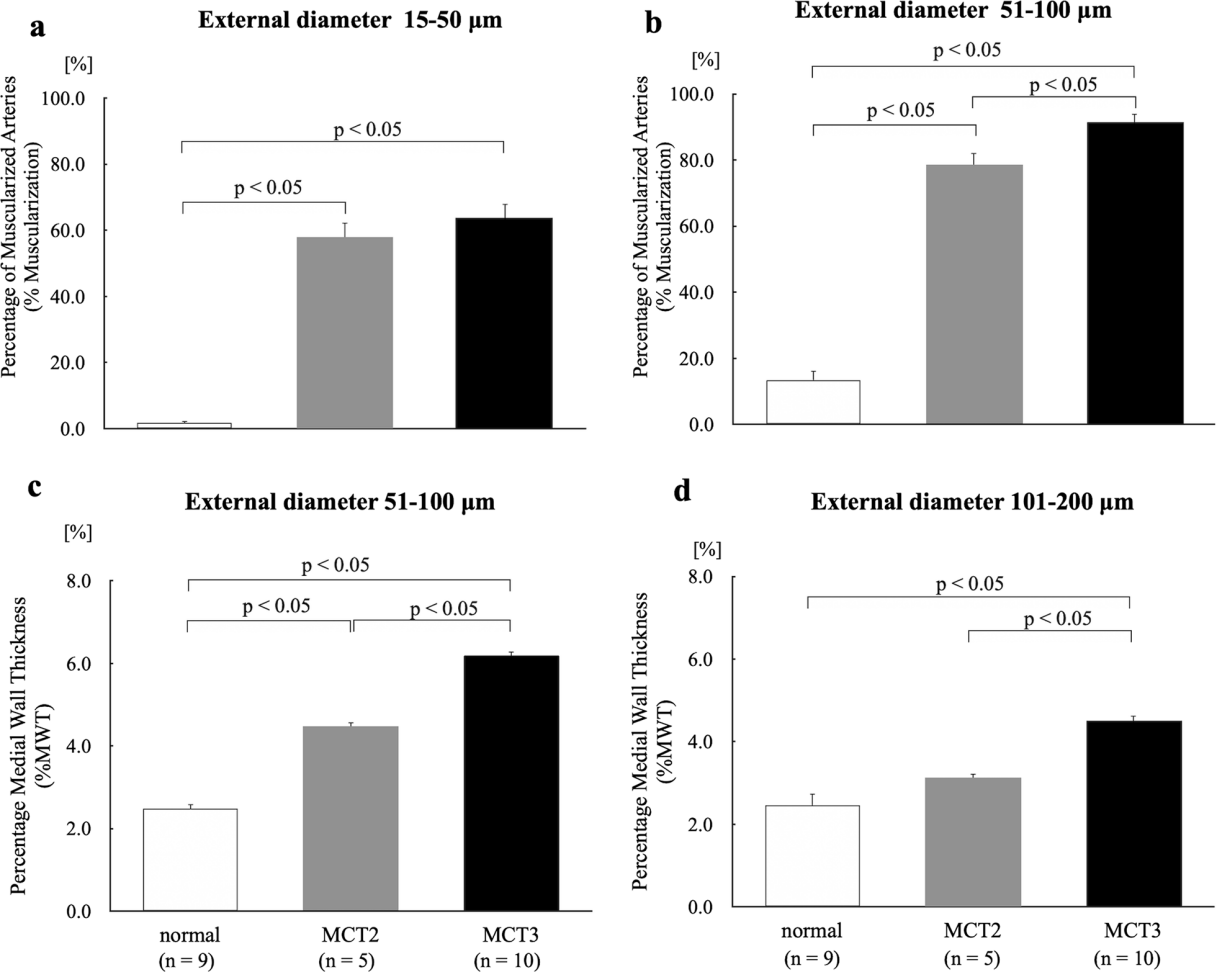
Vascular structural changes at 2 and 3 weeks after monocrotaline injection. **a** Percentage of muscularized arteries (%Muscularization) of peripheral pulmonary arteries with an external diameter of 15–50 μm. **b** %Muscularization of peripheral pulmonary arteries with an external diameter of 51–100 μm. **c** Percentage medial wall thickness (%MWT) of muscularized arteries with an external diameter of 51–100 μm. **d** %MWT of muscularized arteries with an external diameter of 101–200 μm. MCT2: rats at 2 weeks after monocrotaline injection; MCT3: rats at 3 weeks after monocrotaline injection. Bars indicate mean ± standard error. n = number of rats

MCT increased the percentage of muscularized peripheral arteries in the MCT2 and MCT3 groups compared with the normal group (*p* < 0.05) (Fig. 5a). The MCT3 group had higher %Muscularization than the MCT2 group in arteries with an external diameter of 51–100 μm (*p* < 0.05) (Fig. 5b), suggesting increased muscularization in peripheral pulmonary arteries in the MCT3 group.

#### Percentage medial wall thickness

%MWT was increased in the MCT2 and MCT3 groups compared with the normal group in arteries with an external diameter of 51–100 μm (*p* < 0.05) (Fig. 5c). The MCT3 group had higher %MWT than the MCT2 group in arteries with an external diameter of 51–100 μm and 101–200 μm (*p* < 0.05) (Fig. 5c, d), suggesting increased medial wall thickness in the MCT3 group.

### Inflammatory cytokines and chemokines

**Fig. 6:**
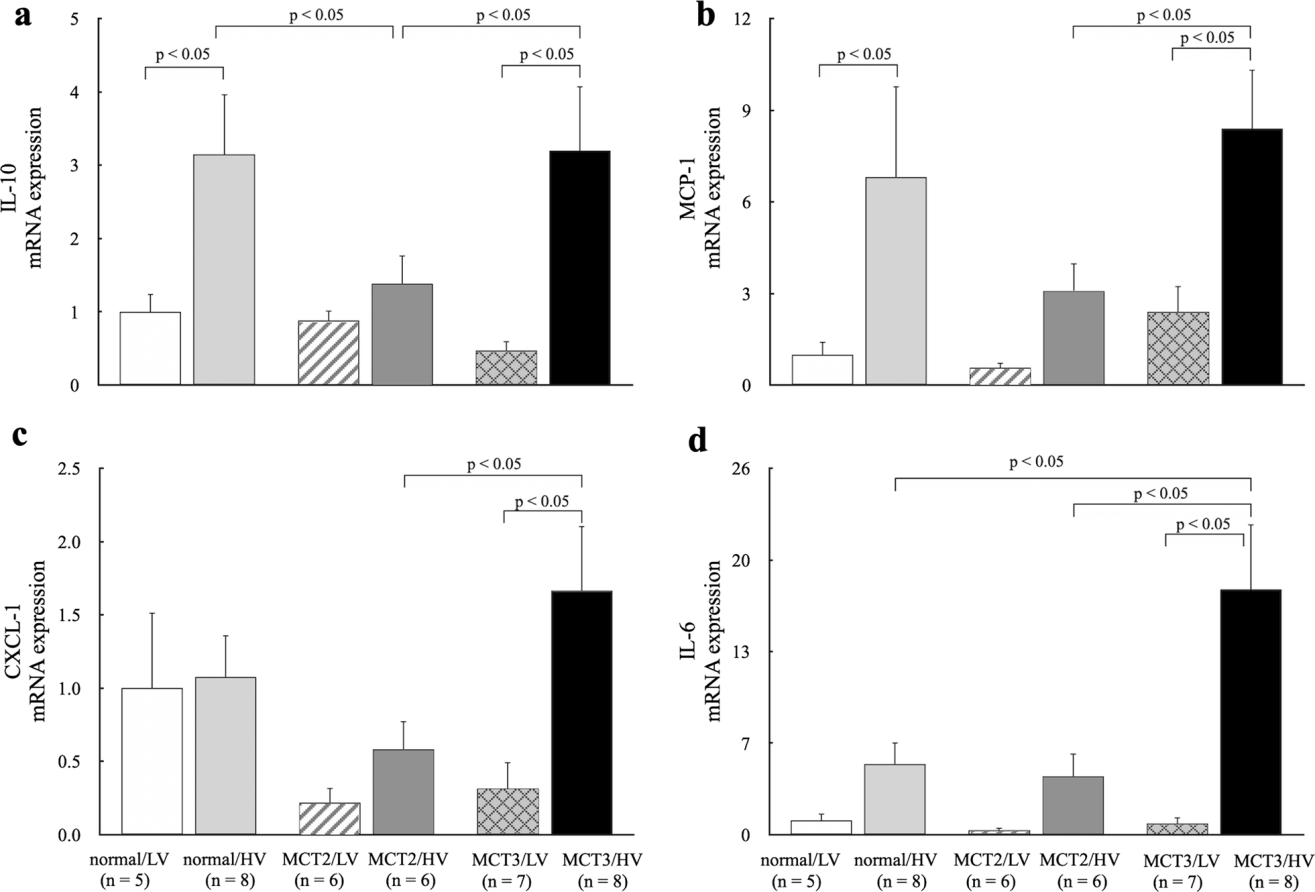
Real-time PCR analysis of cytokine and chemokine mRNA levels in lung tissue in rats. **a** Interleukin (IL)-10. **b** Monocyte chemotactic protein-1 (MCP-1). **c** Chemokine CXCL-1/KC (MIP-1). **d** IL-6. MCT2: rats at 2 weeks after monocrotaline injection; MCT3: rats at 3 weeks after monocrotaline injection; LV: low tidal volume (6 mL/kg); HV: high tidal volume (35 mL/kg). Bars indicate mean ± standard error. n = number of rats

To confirm the accentuated inflammatory response in the HV groups compared with the LV groups, we measured cytokine and chemokine mRNA levels in the lungs (Fig. 6). IL-6, MCP-1, CXCL-1 (MIP-1), and IL-10 mRNA levels were similar among the LV groups. High tidal volume ventilation significantly increased IL-6, MCP-1, CXCL-1 (MIP-1), and IL-10 mRNA levels in MCT3 rats and IL-10 and MCP-1 mRNA levels in normal rats compared with low tidal volume ventilation. There was also a trend toward an increase in the levels of IL-6, MCP-1, CXCL-1 (MIP-1), and IL-10 mRNA levels in MCT2 rats and IL-6 mRNA levels in normal rats compared with low tidal volume ventilation, but the values did not reach statistical significance. These trends were consistent with the insignificant downregulation of IκB, since the decrease in IκB shows the inflammatory reaction. NFκB, which is activated by the decrease in IκB, is located in the upstream of inflammatory cytokines.

### IκB and HMGB-1

**Fig. 7:**
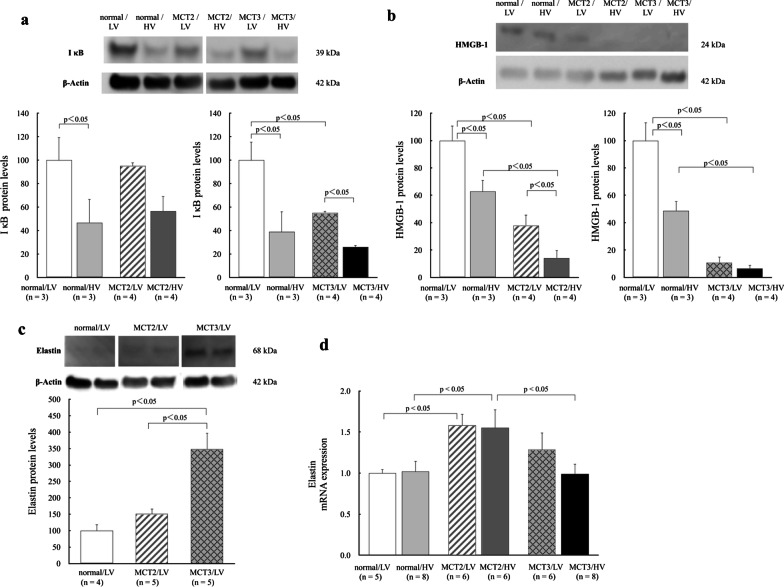
Western blotting analysis of IκB, high-mobility group box 1 (HMGB-1), and elastin in lung tissue. **a** IκB. **b** HMGB-1. **c** Elastin. **d** Elastin mRNA. The lung samples of normal groups were selected randomly from the pooled samples of 9-week-old to 10-week-old rats. MCT2: rats at 2 weeks after monocrotaline injection; MCT3: rats at 3 weeks after monocrotaline injection; LV: low tidal volume (6 mL/kg); HV: high tidal volume (35 mL/kg). Average intensity of normal/LV was taken as 100%. Sample intensity was calculated as a percentage of the average (relative intensity). Bars indicate mean ± standard error. n = number of rats.

As our previous study showed a decrease of IκB protein expression in lungs from MCT-induced PH rats [34] and another study showed that HMGB-1 plasma levels are elevated in PH [38], we determined IκB and HMGB-1 expression in lung tissue and the effect of high tidal volume ventilation on their levels. High tidal volume ventilation significantly decreased IκB protein expression in normal and MCT3 rats (Fig. 7a) and decreased HMGB-1 protein expression in normal and MCT2 rats but not in MCT3 rats (Fig. 7b). The lung samples of normal group rats (Fig. 7a, b) were obtained from 9- to 10-week-old rats. The values of the percentages of lung water content and EBD extravasation were similar between 9-week-old and 10-week-old normal rats. Therefore, in order to reduce the number of experimental animals, the lung samples of normal groups were selected randomly from the pooled samples of 9-week-old to 10-week-old rats.

### Elastin

To investigate the effect of MCT injection on connective tissue protein, lung elastin protein levels were assessed in normal, MCT2, and MCT3 rats ventilated with low tidal volume. Elastin protein expression was significantly increased in MCT3 rats compared with normal and MCT2 rats (Fig. 7c). Elastin mRNA was increased in MCT2 rats compared to normal rats (Fig. 7d). There was no effect on elastin mRNA levels by high tidal volume ventilation (Fig. 7d).

## Discussion

MCT caused PH and hypertensive pulmonary vascular remodeling, and MCT3 rats had significantly higher mPAP, more muscularized peripheral pulmonary arteries, and thicker medial walls of small muscularized arteries than MCT2 rats, showing that the magnitude of PH was severe in MCT3 rats and mild in MCT2 rats. High tidal volume ventilation increased the percentage of lung water content and protein permeability, as measured by EBD extravasation, in normal and PH rats. The deterioration of arterial oxygen tension with high tidal volume ventilation was more attenuated in severe PH (MCT3) rats compared with normal and mild PH (MCT2) rats.

The main focus of this study was VILI in PH rats. Since VILI is associated with the release of inflammatory mediators, the presence of inflammation in the lung before mechanical ventilation might exacerbate VILI. So, the inflammatory component of the present MCT-induced PH model deserve comments, in which reduction of IκBα protein levels were confirmed in the present study. Although the exact mechanism through which MCT causes PH is not known, inflammation is considered to have an important role [35]. Neutrophils have been implicated early in the course of MCT injury [15], and mononuclear cells (particularly macrophages) and lymphocytes infiltrate the alveolar wall by 14 days after injection [32], when plasma and bronchoalveolar lavage fluid MCP-1 levels are also elevated [29]. Our previous study showed that MCT treatment significantly reduces IκBα protein levels in lung tissue, which were restored by treatment with pyrrolidine dithiocarbamate, an NF-κB inhibitor [34]. NF-κB is a transcription factor regulating the expression of genes involved in the inflammatory response. A decrease in the protein levels of IκBα has been reported to be associated with the upregulation of NF-κB activity [39].

The severity of lung injury was similar between normal and mild PH (MCT2) rats with high tidal volume ventilation because there was no difference in the lung wet/dry weight ratio, EBD extravasation, and arterial oxygenation. In contrast, EBD extravasation and arterial oxygenation were less impaired in severe PH (MCT3) rats compared with normal and mild PH (MCT2) rats, suggesting resistance to high tidal volume ventilation-induced lung injury in this group, which was unexpected. We had anticipated that lung injury caused by high tidal volume ventilation might be more severe in MCT-induced PH rats compared with normal rats, and that MCT3 rats would have more severe lung injury than MCT2 rats, because MCT-induced PH is a type of inflammatory PH and inflammatory cytokines are involved in the development of VILI [7, 8, 28–30]. However, high tidal volume ventilation-induced EBD extravasation was lower in PH lungs than in normal lungs and we could not detect significant differences in arterial oxygenation between high and low tidal volume ventilation in severe PH rats. It seemed that hypertensive vascular changes may be associated with protection against the development of VILI, regardless of the presence of inflammation.

High tidal volume ventilation tended to increase the expression of proinflammatory cytokines in normal and PH rats, suggesting an increased inflammatory response with high tidal volume ventilation, which is consistent with earlier reports [1, 2, 7–10]. Under high tidal volume ventilation, severe PH (MCT3) rats had significantly higher cytokine expression, such as IL-6, IL-10, MCP-1, and CXCL-1, than mild PH (MCT2) rats, showing that the high tidal volume ventilation-induced inflammatory response was accentuated in MCT3 rats compared with MCT2 rats. According to this line of thought, MCT3 rats were expected to have more severe lung damage with high tidal volume ventilation, but this was not the case. Although the inflammatory component was high in MCT3 rats, arterial oxygenation and EBD permeability were less impaired. Because mechanical trauma (volutrauma) and inflammation (biotrauma) additively or synergistically cause ventilator-associated lung injury [2], the mechanical trauma component might be reduced in MCT3 rats due to hypertensive vascular remodeling. The increase in transmural and transpulmonary pressure, that is, mechanical forces, during high tidal volume ventilation can cause breaks in the capillary endothelial layer and alveolar epithelium [40], which is mechanical damage. The thick vascular wall in PH might be less vulnerable to mechanical force compared with normal vasculature. Similar results were observed in another rat model of PH, namely, chronic hypoxia-induced PH, and the authors suggested that the enhanced tensile strength associated with pulmonary vascular remodeling would protect against VILI [27].

Overinflation and the increase in surface forces induced by high tidal volume ventilation both lead to an augmentation of vascular transmural pressure [41], partly leading to the formation of hydrostatic-type edema. Thus, further vascular transmural pressure would be added in the presence of PH under high tidal volume ventilation in normal lung vasculature, thereby inducing lung edema. However, in the PH lung with vascular remodeling, the increase of transmural pressure during high tidal volume ventilation might be less compared with the normal lung owing to the presence of thick-walled vessels with increased stiffness and decreased lung compliance [15, 21], which might reduce the effect of hyperinflation. HV-induced lung edema was comparable among normal, mild PH and severe PH rats, which was evaluated by lung the percentage of lung water content (wet lung weight − dried lung weight)/(wet lung weight) × 100) in the present study (Fig. 2b). This method was probably insensitive to detect the increased pulmonary permeability than EBD extravasation, since EBD extravasation during HV ventilation was less in mild and severe PH rats compared to normal rats (Fig. 2a). EBD extravasation is more sensitive for detecting the increase in protein permeability in the lung than dry/wet weight ratios in the early phase of VILI [11].

HMGB-1 is normally present as a nuclear protein and is passively released from damaged cells [41]. The reduction of HMGB-1 expression in lung tissue from rats with high tidal volume ventilation, consistent with a previous study [11], might be due to the increased release of HMGB-1 protein into the circulation [42, 43], which might reflect cell damage. Because high tidal volume ventilation caused a significant decrease of HMGB-1 expression in MCT2 rats, but not in MCT3 rats, this might suggest that the magnitude of cell damage due to high tidal volume ventilation might be less in MCT3 rats.

Given that elevated vascular wall stress in PH increases the production and accumulation of elastin [44], we determined the expression of elastin in lung tissue and showed that MCT3 rats had significantly higher lung elastin levels than normal and MCT2 rats. Regardless of the etiology, hypertensive pulmonary vascular remodeling includes new muscularization of normally non-muscularized peripheral pulmonary arteries, medial wall hypertrophy of muscularized arteries, and increased vascular connective tissue protein levels such as collagen and elastin [20, 22]. These changes increase vascular stiffness. Arterial stiffness can be increased by an increase in the expression of rigid wall materials such as collagen, elastin, fibronectin, and proteoglycans [15, 45], which was evidenced by the higher levels of lung elastin observed in MCT3 rats by western blotting in the present study. Smooth muscle cell hypertrophy and hyperplasia are associated with the increased production of collagen because these cells produce connective tissues such as collagen and elastin [15, 22, 31]. In thick-walled pulmonary arteries, a process of degradation and increased synthesis of connective tissue protein in the subendothelium and media occurs [31]. The width of the elastic lamina and volume of collagen are increased in MCT-injected rats compared with non-treated rats [31]. In the present study, hypertensive pulmonary vascular changes were clearly more accentuated in severe PH (MCT3) rats compared with mild PH rats (MCT2), which possibly resulted in the increased vascular stiffness. This increased stiffness might lead to protection from the increased pressure outside of the vasculature associated with lung overinflation. In normal vasculature, lung inflation flattens the peripheral pulmonary vasculature, raising the tension of the vasculature [46], which might be reduced in hypertensive vasculature because of increased stiffness. PIP after the start of high tidal volume ventilation was higher in PH rats than in normal rats, suggesting decreased lung compliance in PH rats, where severe PH (MCT3) rats had less lung compliance than mild PH (MCT2) rats. An increase in connective tissue protein levels in the PH lung might decrease compliance [21].

## Limitations

The first limitation of this study is that it was observational and not mechanistic. Although observational, this study suggested a relationship between hypertensive pulmonary vascular remodeling and the occurrence of VILI. Most of the previous studies have used control rats and/or rats treated with HCl [4], lipopolysaccharides [5], alpha-naphtylthiourea [3], and 100% O_2_ [11], which are acute models without hypertensive vascular remodeling. Second, the MCT model does not completely recapitulate human pulmonary arterial hypertension. However, the most commonly used animal PH models are the chronic hypoxia-induced PH model and MCT-PH model; the former as a model of PH in patients residing at high altitude and those with chronic obstructive pulmonary disease, and the latter as a model of pulmonary arterial hypertension [18, 19] and inflammation-related PH. Because an earlier study demonstrated that vascular remodeling is protective against the development of VILI in ventilated chronic hypoxia-induced PH rats with high tidal volume [27], the present results in the MCT model were similar to those in hypoxia-induced PH. Thus, taking these results into consideration, we could say that vascular remodeling might be protective against the effect of injurious mechanical ventilation, regardless of the etiology of PH. Third, the magnitude of protection might be subtle given that we could not detect a significant decrease in the percentage of lung water content. However, lung permeability and arterial oxygenation were less deteriorated in severe PH rats. The percentage of lung water content might be less sensitive than EBD extravasation [11]. Fourth, although hypertensive vasculature might be protective against mechanical force to the lung itself, the systemic effect of inflammatory cytokines from the PH lung might cause remote organ failure, such as the kidney and liver because VILI is associated with or causes multiple organ dysfunction [1]. Further investigations are necessary to determine the presence of the dysfunction of remote organs other than the lung in PH rats. It is of great interest whether use of HV improves or worsens the prognosis of PH since the expression of genes involved in the inflammatory response in the lung was changed after HV. Thus, mean pulmonary arterial pressure and pulmonary vascular remodeling 1–2 weeks after HV would deserve investigation. Whether or not HV worsens the prognosis of PH might be judged by examining the survival rate until 5 weeks with time course.

## Conclusion

In summary, the results reported here and those from a previous study [27] showed that hypertensive pulmonary vascular structural changes might protect against the occurrence of VILI, which might attenuate the magnitude of lung injury.

## Supplementary Information

**Additional file 1.** The original gels in Fig. 7a, 7b, and 7c.

## Data Availability

All original data will be made available upon from the first author (m.kawai@suzuka-u.ac.jp) reasonable request.

## Abbreviations

PH: Pulmonary hypertension
MCT: Monocrotaline
MCT2: Rats at 2 weeks after monocrotaline injection
MCT3: Rats at 3 weeks after monocrotaline injection
LV: Low tidal volume (6 mL/kg)
HV: High tidal volume (35 mL/kg)
PaO_2_: Arterial oxygen pressure
EBD: Evans blue dye
MWT: Medial wall thickness
IL: Interleukin (IL)
CXCL-1: C-X-C motif chemokine ligand 1
TNF: Tumor necrosis factor
MCP: Monocyte chemotactic protein
VILI: Ventilator-induced lung injury
PIP: Peak inspiratory airway pressure
mPAP: Mean pulmonary arterial pressure
RVH: Right ventricular hypertrophy
RV/LV + S: Right ventricular weight/left ventricular plus septal weight
HMGB-1: High-mobility group box 1
