# Supplementary material for: Lung damage created by high tidal volume ventilation in rats with monocrotaline-induced pulmonary hypertension
Source: BMC Pulm Med. 2022 Mar 5;22:78. doi: 10.1186/s12890-022-01867-6 (PMC8897872; doi:10.1186/s12890-022-01867-6)
Supplement: Supplementary file 1 — Additional file 1. The original gels in Fig. 7a, 7b, and 7c. [file 12890_2022_1867_MOESM1_ESM.pdf]

Supplementary Western blot image

Figure S7 a

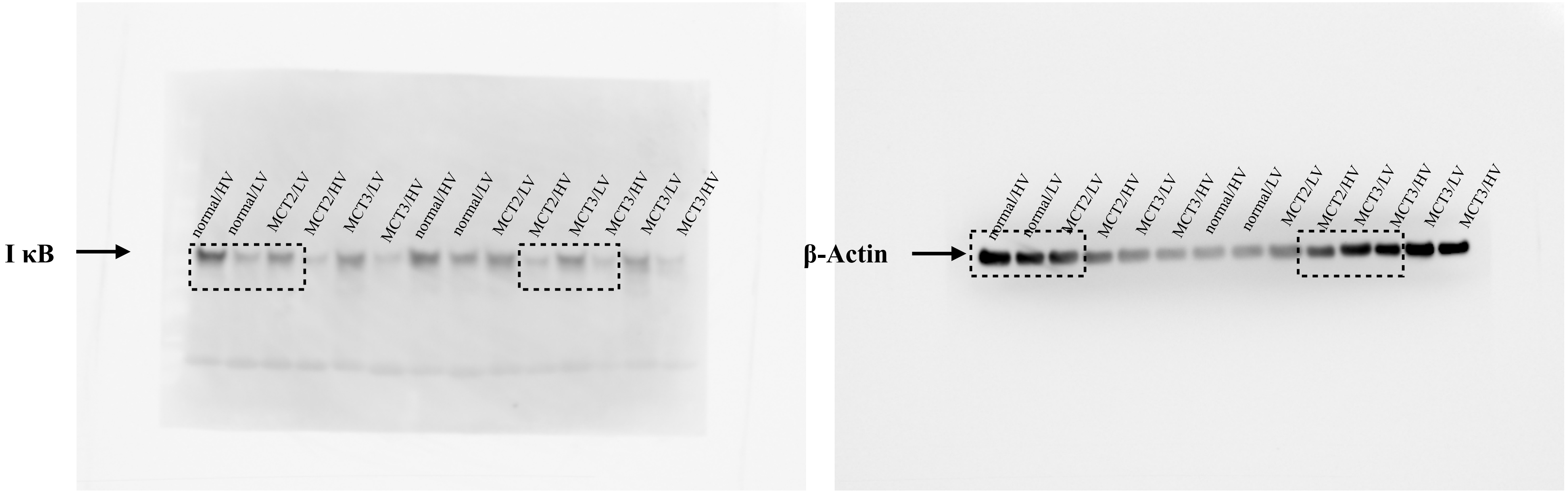

Figure S7 a. Western blotting analysis of I ϰB in lung tissue. Full length blot for Western blot images used in Fig. 7a. Black dotted squares indicate the representative blots in the main figures.
MCT2: rats at 2 weeks after monocrotaline injection; MCT3: rats at 3 weeks after monocrotaline injection; LV: low tidal volume (6 mL/kg); HV: high tidal volume (35 mL/kg).

Figure S7 b

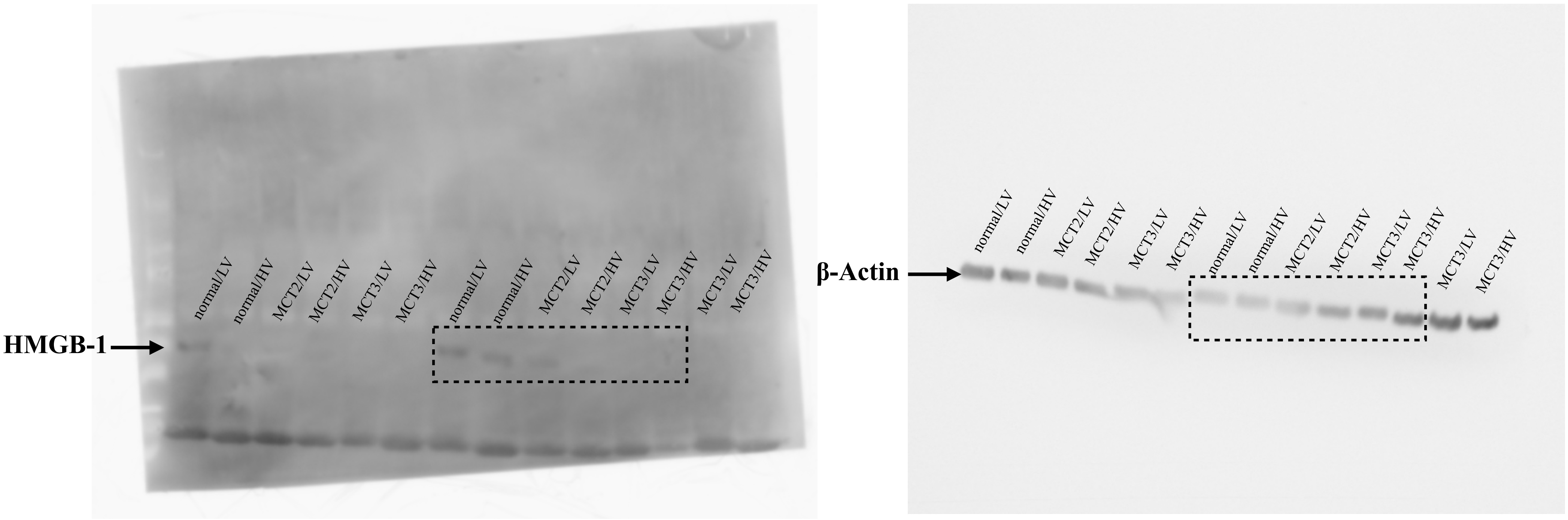

Figure S7 b. Western blotting analysis of HMBG-1 in lung tissue. Full length blot for Western blot images used in Fig. 7b. Black dotted squares indicate the representative blots in the main figures.
MCT2: rats at 2 weeks after monocrotaline injection; MCT3: rats at 3 weeks after monocrotaline injection; LV: low tidal volume (6 mL/kg); HV: high tidal volume (35 mL/kg).

Figure S7 c

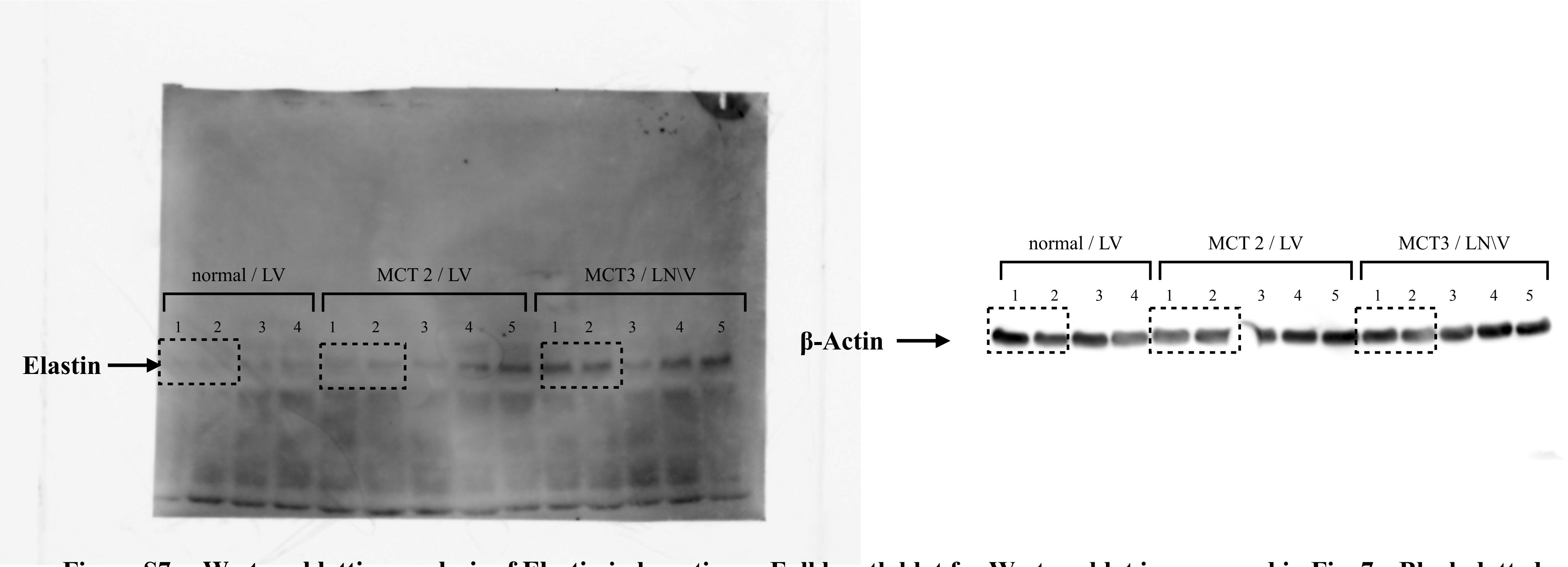

Figure S7 c. Western blotting analysis of Elastin in lung tissue. Full length blot for Western blot images used in Fig. 7c. Black dotted squares indicate the representative blots in the main figures.
MCT2: rats at 2 weeks after monocrotaline injection; MCT3: rats at 3 weeks after monocrotaline injection; LV: low tidal volume (6 mL/kg); HV: high tidal volume (35 mL/kg).
